# Supplementary material for: Integration of Response Surface Methodology (RSM) and Principal Component Analysis (PCA) as an Optimization Tool for Polymer Inclusion Membrane Based-Optodes Designed for Hg(II), Cd(II), and Pb(II)
Source: Membranes (Basel). 2021 Apr 14;11(4):288. doi: 10.3390/membranes11040288 (PMC8070702; doi:10.3390/membranes11040288)
Supplement: Supplementary file 1 [file membranes-11-00288-s001.pdf]

membranes

MDPI

Supporting Information

Integration of response surface methodology (RSM) and principal component analysis (PCA) as an optimization tool of polymer inclusion membrane based-optodes designed for Hg(II), Cd(II) and Pb(II)

Jeniffer García-Beleño, Eduardo Rodríguez de San Miguel*

Departamento de Química Analítica, Facultad de Química, Universidad Nacional Autónoma de México (UNAM), Ciudad Universitaria, 04510, Ciudad de México, México; jgarciab@comunidad.unam.mx

* Correspondence: erdsmg@unam.mx

Table S1. Values of the predicted desirability of the multivariate analysis performed with the M1 process method, using Dz as chromophore.

Experimental runs	Response		
	Expected Desirability		
	Dz – Hg(II)	Dz – Cd(II)	Dz – Pb(II)
1	0.587493	0.412899	0.583719
2	0.587493	0.412899	0.583719
3	0.47074	0.239581	0.0
4	0.557221	0.351363	0.494072
5	0.467964	0.384996	0.447025
6	0.587493	0.412899	0.583719
7	0.639942	0.614606	0.583512
8	0.61091	0.451523	0.580387
9	0.498601	0.550099	0.515556
10	0.639942	0.462867	0.476102
11	0.61091	0.469964	0.548087
12	0.705562	0.526763	0.444928
13	0.47074	0.180432	0.313694
14	0.519043	0.294723	0.535386
15	0.519043	0.242642	0.609567
16	0.557221	0.365713	0.482335
17	0.628188	0.504092	0.655115
18	0.587493	0.440114	0.583719
19	0.634034	0.364699	0.39621
20	0.628188	0.320985	0.799071
21	0.587493	0.383758	0.583719
22	0.587493	0.412899	0.583719

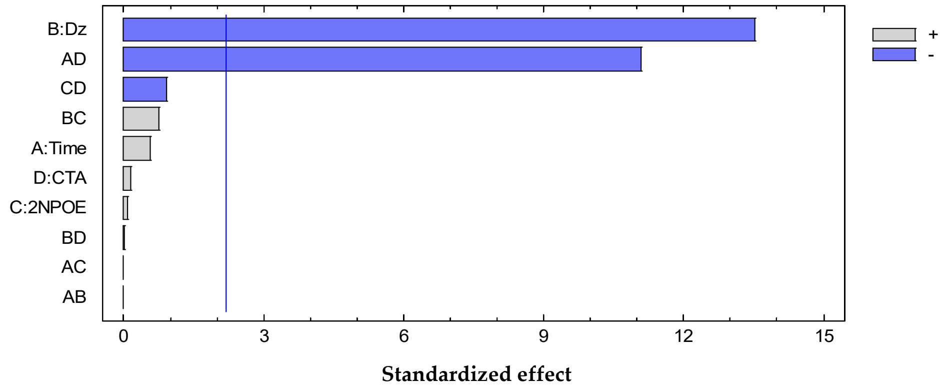

Figure S1. Pareto of the multivariate analysis performed with the M1 process method for the system Dz – Hg(II).

Table S2. ANOVA values of the multivariate analysis performed with the M1 process method for the system Dz – Hg(II).

Source	*Sum of squares*	*Df*	*Mean square*	*F-Ratio*	*P-Value*
**A:Time**	0,0000836037	1	0,0000836037	0,33	0,5759
**B:Dz**	0,0460006	1	0,0460006	182,85	**0,0000**
**C:2NPOE**	0,0000018768	1	0,0000018768	0,01	0,9327
**D:CTA**	0,00000643161	1	0,00000643161	0,03	0,8759
**AB**	0,0	1	0,0	0,00	1,0000
**AC**	0,0	1	0,0	0,00	1,0000
**AD**	0,0309215	1	0,0309215	122,91	**0,0000**
**BC**	0,0001458	1	0,0001458	0,58	0,4625
**BD**	1,26293E-7	1	1,26293E-7	0,00	0,9825
**CD**	0,000213563	1	0,000213563	0,85	0,3766
**Total Error**	0,00276736	11	0,000251578		
**Total (corrected)**	0,0840181	21			
**R^2**	96,7062 %				
**Adj - R^2**	93,7119 %				
**Standard error**	0,0158612				
**Std. Dev**	0,00881342				

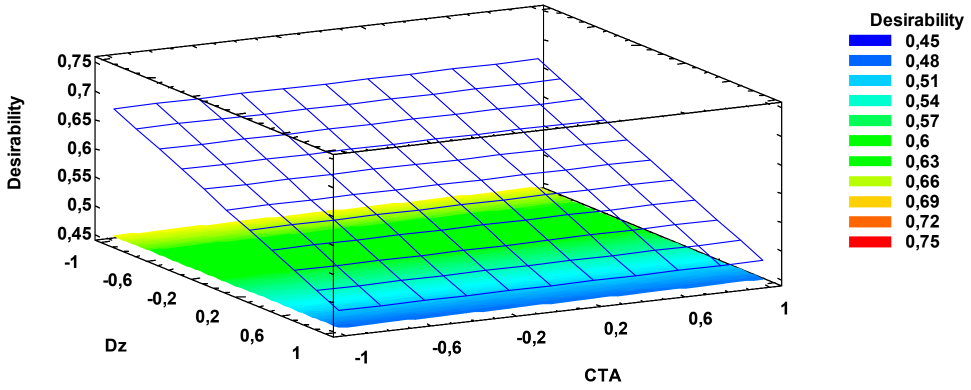

Figure S2. Response surface and contour plots of the multivariate analysis performed with the M1 process method for the system Dz – Hg(II), when 2NPOE = Time = 0,0.

For the Dz – Hg(II) system the model was:

D = 0.576507 - 0.09592*Dz - 0.235797*Time*CTA (S1)

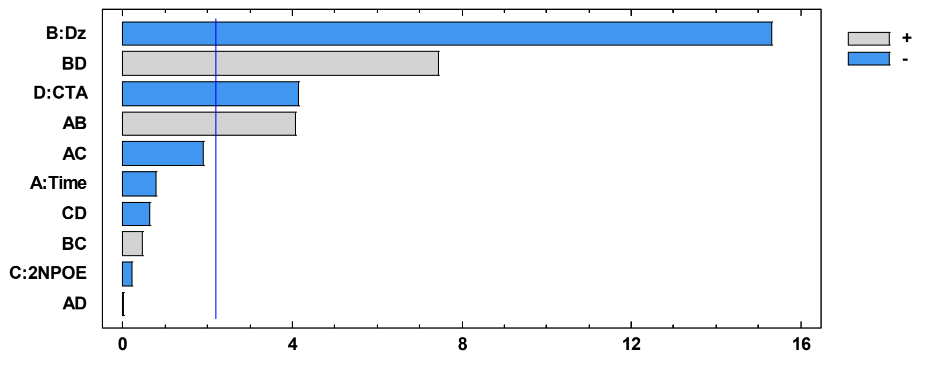

Figure S3. Pareto of the multivariate analysis performed with the M1 process method for the system Dz – Cd(II).

Table S3. ANOVA values of the multivariate analysis performed with the M1 process method for the system Dz – Cd(II).

Source	*Sum of squares*	*Df*	*Mean square*	*F-Ratio*	*P-Value*
**A:Time**	0,00041896	1	0,00041896	0,62	0,4469
**B:Dz**	0,158043	1	0,158043	234,68	**0,0000**
**C:2NPOE**	0,0000281747	1	0,0000281747	0,04	0,8417
**D:CTA**	0,0116151	1	0,0116151	17,25	**0,0016**
**AB**	0,0111184	1	0,0111184	16,51	**0,0019**
**AC**	0,00239427	1	0,00239427	3,56	0,0860
**AD**	2,15028E-7	1	2,15028E-7	0,00	0,9861
**BC**	0,00013992	1	0,00013992	0,21	0,6574
**BD**	0,0372474	1	0,0372474	55,31	**0,0000**
**CD**	0,000279159	1	0,000279159	0,41	0,5329
**Total Error**	0,00740776	11	0,000673432		
**Total (corrected)**	0,230575	21			
**R^2**	96,7873 %				
**Adj - R^2**	93,8666 %				
**Standard error**	0,0259506				
**Std. Dev**	0,0136648				

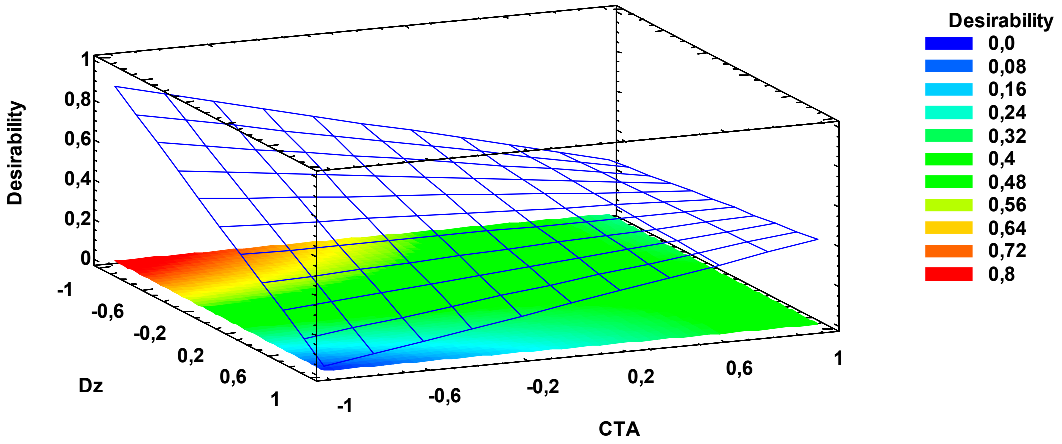

Figure S4. Response surface and contour plots of the multivariate analysis performed with the M1 process method for the system Dz – Cd(II), when 2NPOE = Time = 0,0.

For the Dz – Cd(II) system, the equations model was:

D = 0.400003 - 0.177793*Dz - 0.0481715*CTA + 0.12176*Time*Dz + 0.251488*Dz*CTA (S2)

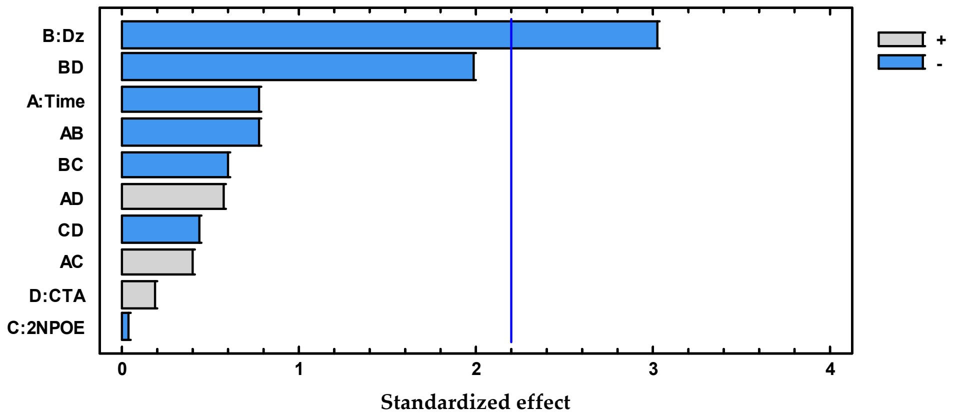

Figure S5. Pareto of the multivariate analysis performed with the M1 process method for the system Dz – Pb(II).

Table S4. ANOVA values of the multivariate analysis performed with the M1 process method for the system Dz – Pb(II).

Source	*Sum of squares*	*Df*	*Mean square*	*F-Ratio*	*P-Value*
**A:Time**	0,0106482	1	0,0106482	0,60	0,4558
**B:Dz**	0,162817	1	0,162817	9,14	**0,0116**
**C:2NPOE**	0,0000254771	1	0,0000254771	0,00	0,9705
**D:CTA**	0,000659052	1	0,000659052	0,04	0,8510
**AB**	0,0106383	1	0,0106383	0,60	0,4560
**AC**	0,00286615	1	0,00286615	0,16	0,6960
**AD**	0,0058389	1	0,0058389	0,33	0,5785
**BC**	0,00634343	1	0,00634343	0,36	0,5628
**BD**	0,0699709	1	0,0699709	3,93	0,0731
**CD**	0,00349611	1	0,00349611	0,20	0,6664
**Total Error**	0,195983	11	0,0178166		
**Total (corrected)**	0,480365	21			
**R^2**	59,2012 %				
**Adj - R^2**	22,1114 %				
**Standard error**	0,133479				
**Std. Dev**	0,0646804				

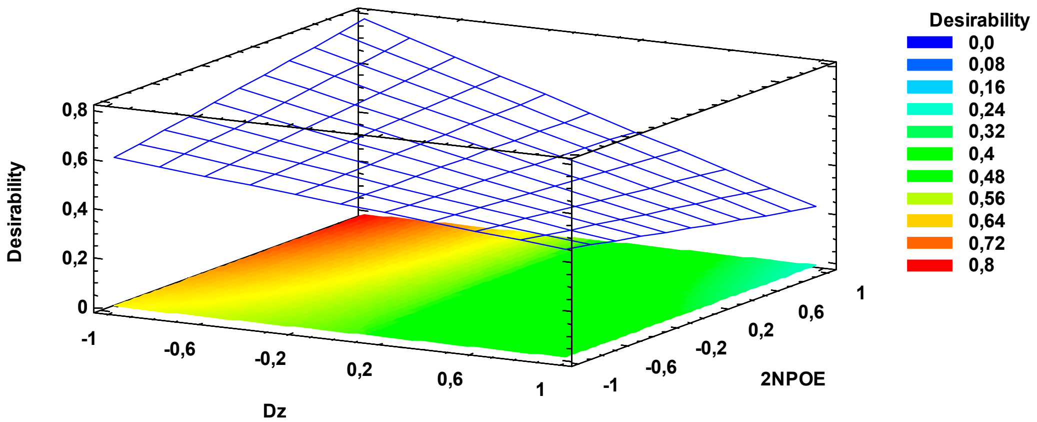

Figure S6. Response surface and contour plots of the multivariate analysis performed with the M1 process method for the system Dz – Pb(II), when CTA = Time = 0,0.

For the system Dz – Pb(II) the model was:

D = 0.517454 - 0.046148*Time - 0.180459*Dz (S3)

Table S5. Values of the predicted desirability of the multivariate analysis performed with the M1 process method using PAN as chromophore.

Experimental runs	Response		
	Expected Desirability		
	PAN – Cd(II)	PAN – Pb(II)	PAN – Hg(II)
1	0.412899	0.706786	0.554163
2	0.412899	0.706786	0.554163
3	0.239581	0.791831	0.531357
4	0.351363	0.770515	0.554544
5	0.384996	0.628514	0.568758
6	0.412899	0.706786	0.554163
7	0.614606	0.489938	0.503729
8	0.451523	0.663153	0.545871
9	0.550099	0.491027	0.367459
10	0.462867	0.343253	0.503729
11	0.469964	0.61407	0.545871
12	0.526763	0.454684	0.367459
13	0.180432	0.554761	0.531357
14	0.294723	0.739257	0.547071
15	0.242642	0.620121	0.475916
16	0.365713	0.713486	0.554544
17	0.504092	0.544041	0.52937
18	0.440114	0.554518	0.442246
19	0.364699	0.581995	0.568758
20	0.320985	0.3661	0.800616
21	0.383758	0.554518	0.647003
22	0.412899	0.706786	0.554163

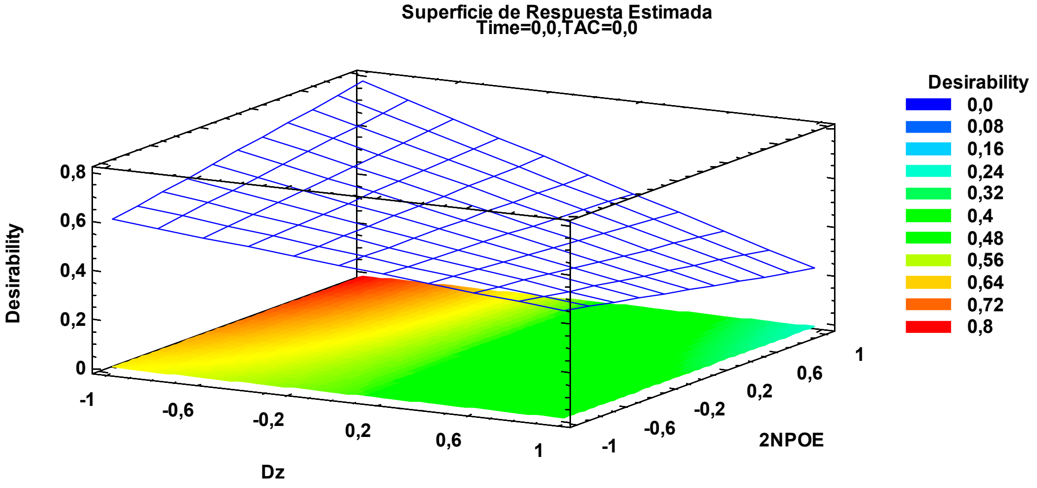

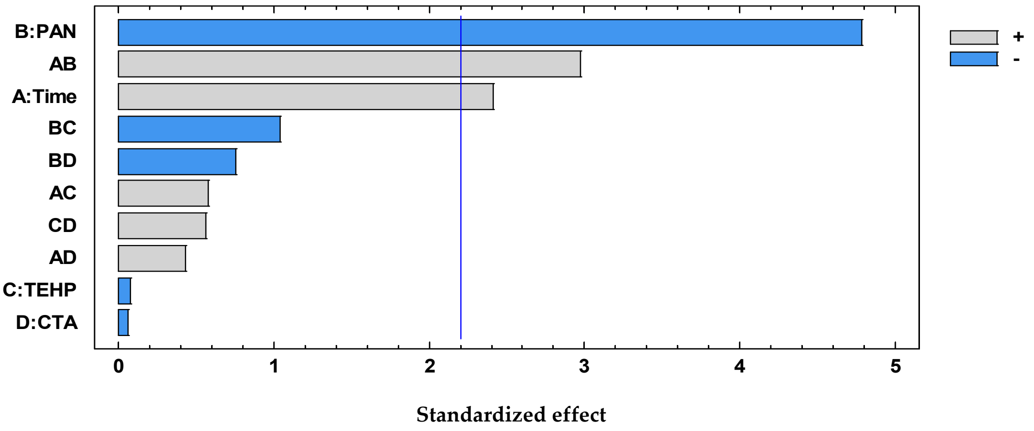

Figure S7. Pareto of the multivariate analysis performed with the M2 process method for the system PAN – Cd(II).

Table S5. ANOVA values of the multivariate analysis performed with the M1 process method for the system PAN – Cd(II).

Source	*Sum of squares*	*Df*	*Mean square*	*F-Ratio*	*P-Value*
**A:Time**	0,0274138	1	0,0274138	5,82	**0,0344**
**B:PAN**	0,107617	1	0,107617	22,86	**0,0006**
**C:THEP**	0,0000307475	1	0,0000307475	0,01	0,9370
**D:CTA**	0,0000184635	1	0,0000184635	0,00	0,9512
**AB**	0,0415196	1	0,0415196	8,82	**0,0127**
**AC**	0,0015598	1	0,0015598	0,33	0,5764
**AD**	0,000867428	1	0,000867428	0,18	0,6760
**BC**	0,00509963	1	0,00509963	1,08	0,3203
**BD**	0,00270399	1	0,00270399	0,57	0,4644
**CD**	0,00150323	1	0,00150323	0,32	0,5833
**Total Error**	0,0517731	11	0,00470665		
**Total (corrected)**	0,264118	21			
**R^2**	80,3977 %				
**Adj - R^2**	62,5774 %				
**Standard error**	0,068605				
**Std. Dev**	0,0320632				

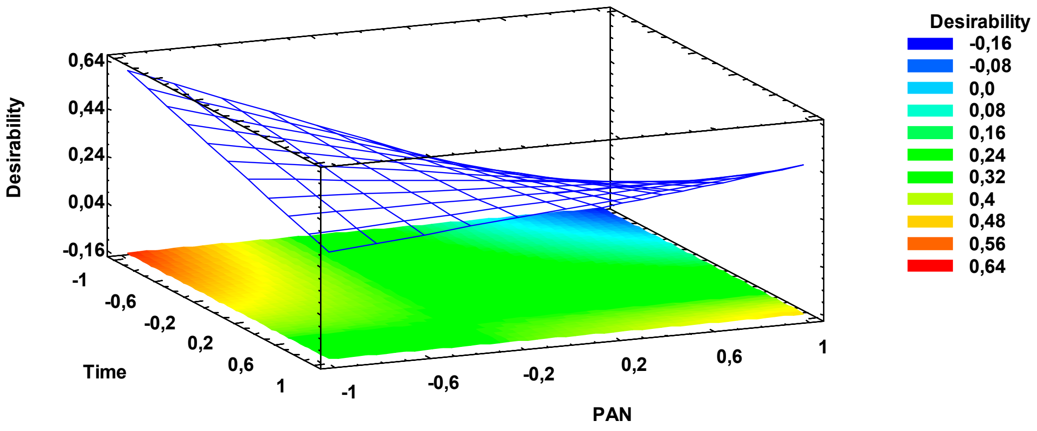

Figure S8. Response surface and contour plots of the multivariate analysis performed with the M1 process method for the system PAN – Cd(II), when CTA = Time = 0,0.

For the system PAN – Cd(II) the model was:

$$D = 0.3001 + 0.0740457*Time - 0.146713*PAN + 0.235293*Time*PAN \quad (S4)$$

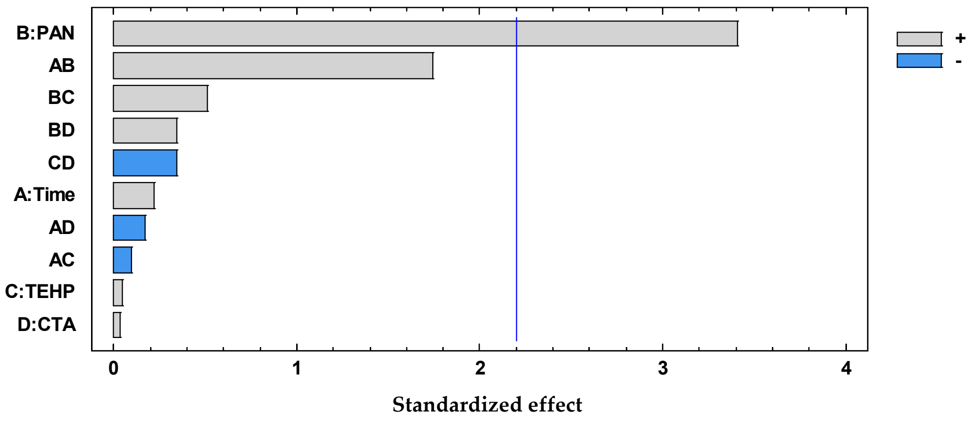

Figure S9. Pareto of the multivariate analysis performed with the M2 process method for the system PAN – Pb(II).

Table S6. ANOVA values of the multivariate analysis performed with the M1 process method for the system PAN – Pb(II).

Source	*Sum of squares*	*Df*	*Mean square*	*F-Ratio*	*P-Value*
**A:Time**	0,000588688	1	0,000588688	0,05	0,8297
**B:PAN**	0,14116	1	0,14116	11,63	**0,0058**
**C:THEP**	0,0000255253	1	0,0000255253	0,00	0,9642
**D:CTA**	0,0000136384	1	0,0000136384	0,00	0,9739

**AB**	0,036817	1	0,036817	3,03	0,1095
**AC**	0,00010842	1	0,00010842	0,01	0,9264
**AD**	0,000350622	1	0,000350622	0,03	0,8681
**BC**	0,00317229	1	0,00317229	0,26	0,6193
**BD**	0,00142016	1	0,00142016	0,12	0,7388
**CD**	0,00141152	1	0,00141152	0,12	0,7395
**Total Error**	0,133535	11	0,0121396		
**Total (corrected)**	0,324055	21			
**R^2**	58,7924 %				
**Adj - R^2**	21,3309 %				
**Standard error**	0,11018				
**Std. Dev**	0,0749911				

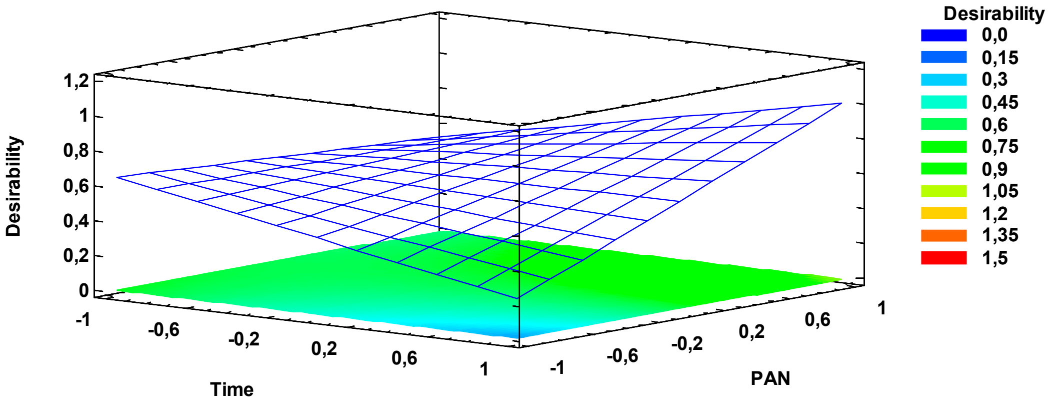

Figure S10. Response surface and contour plots of the multivariate analysis performed with the M1 process method for the system PAN – Pb(II), when CTA = Time = 0,0.

For the system PAN – Pb(II) the model was:

D = 0.604665 + 0.168028*PAN (S5)

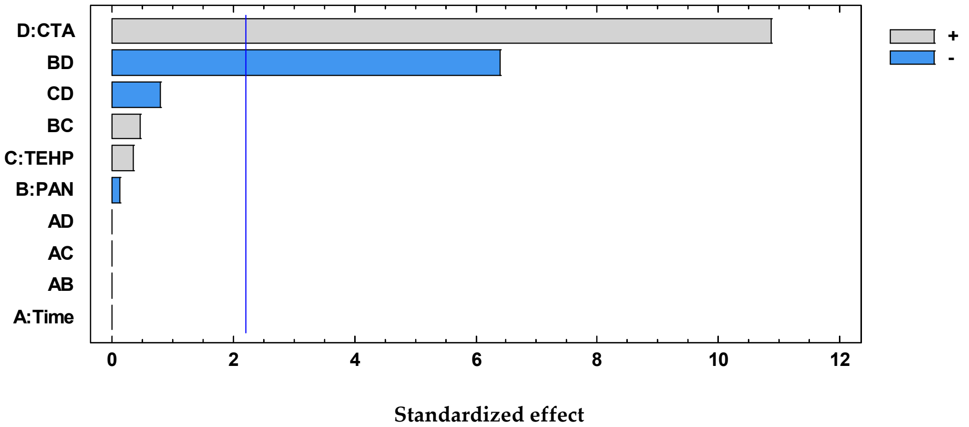

Figure S11. Pareto of the multivariate analysis performed with the M1 process method for the system PAN – Hg(II).

Table S7. ANOVA values of the multivariate analysis performed with the M1 process method for the system PAN – Hg(II).

Source	*Sum of squares*	*Df*	*Mean square*	*F-Ratio*	*P-Value*
**A:Time**	0,0	1	0,0	0,00	1,0000
**B:PAN**	0,000013335	1	0,000013335	0,01	0,9062
**C:THEP**	0,000103831	1	0,000103831	0,11	0,7429
**D:CTA**	0,108594	1	0,108594	118,34	**0,0000**
**AB**	0,0	1	0,0	0,00	1,0000
**AC**	0,0	1	0,0	0,00	1,0000
**AD**	0,0	1	0,0	0,00	1,0000
**BC**	0,000192123	1	0,000192123	0,21	0,6562
**BD**	0,0376356	1	0,0376356	41,01	**0,0001**
**CD**	0,000583345	1	0,000583345	0,64	0,4421
**Total Error**	0,010094	11	0,00091764		
**Total (corrected)**	0,158191	21			
**R^2**	93,6191 %				
**Adj – R^2**	87,8183 %				
**Standard error**	0,0302926				
**Std. Dev**	0,0176483				

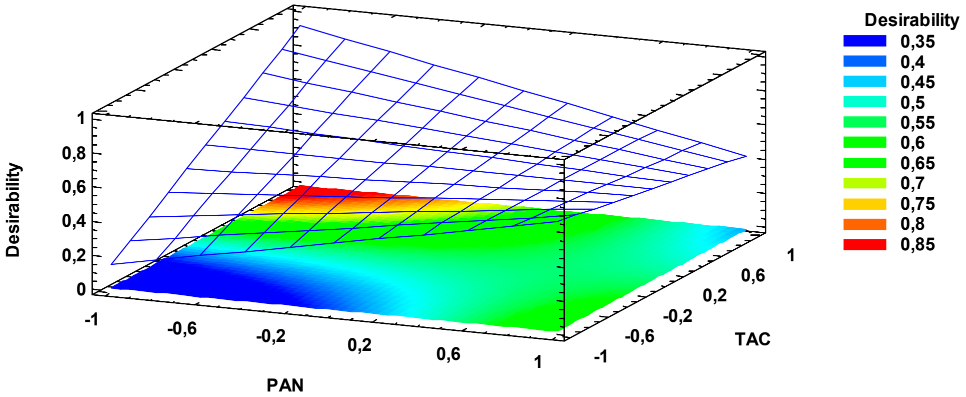

Figure S12. Response surface and contour plots of the multivariate analysis performed with the M1 process method for the system PAN – Hg(II), when CTA = Time = 0,0.

For the system PAN – Hg(II) the model was:

Desirability = 0.536483 + 0.147293*CTA - 0.252795*PAN*CTA (S6)

Table S8. Optimization results using the score values of the first two principal components of PCA for membranes after complexation.

<table>
<tr><th rowspan="2"></th><th rowspan="2">Optimal experiment</th><th colspan="2">Appearance of the membrane</th><th rowspan="2">Spectral</th></tr>
<tr><th>Before</th><th>After</th></tr>
<tr><td>PAN + Hg</td><td>20</td><td>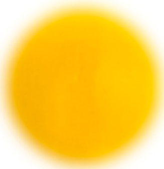</td><td>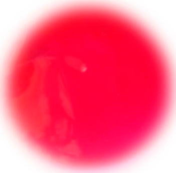</td><td>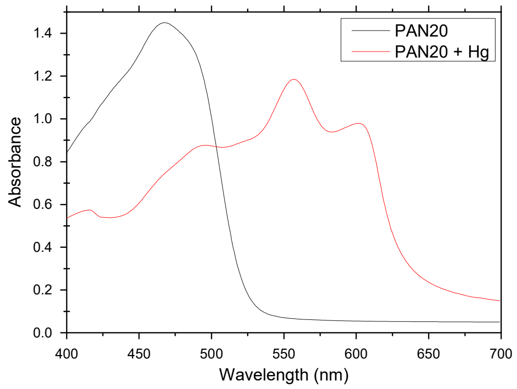
</td></tr>
<tr><td>PAN + Cd</td><td>7</td><td>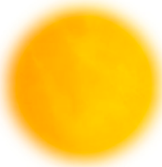</td><td>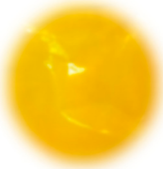</td><td>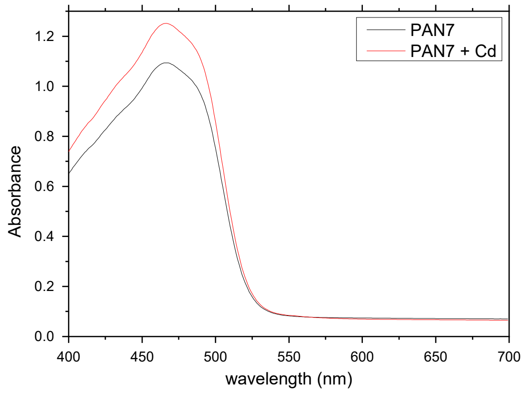
</td></tr>
<tr><td>PAN + Pb</td><td>3</td><td>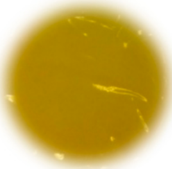</td><td>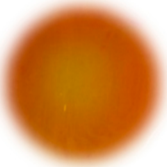</td><td>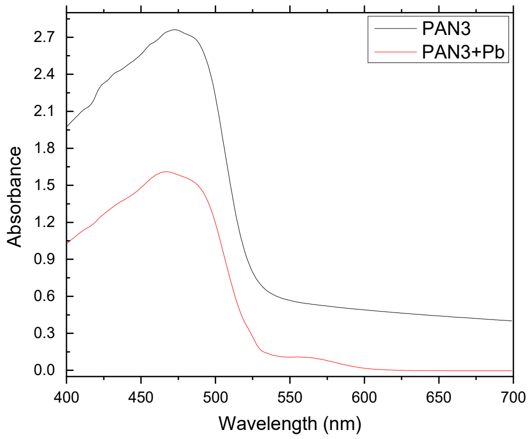
</td></tr>
</table>

**Dz + Hg**	12	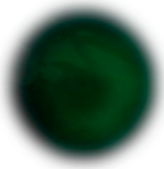	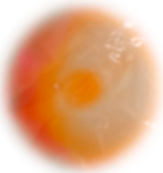	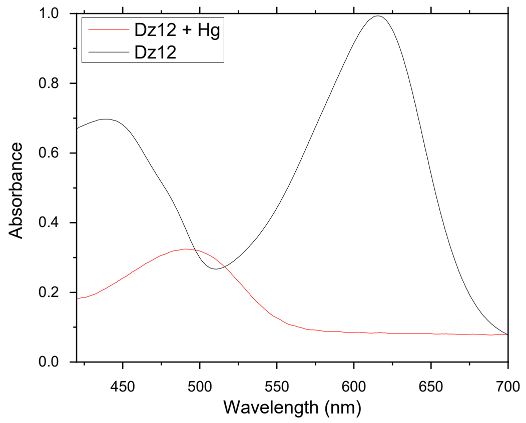
**Dz + Cd**	7	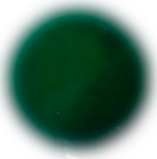	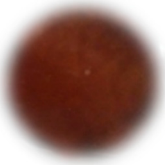	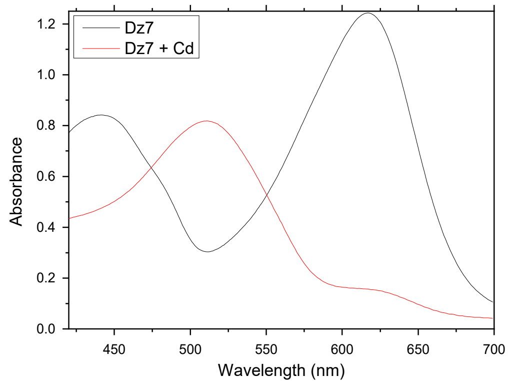
**Dz + Pb**	20	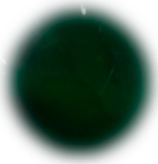	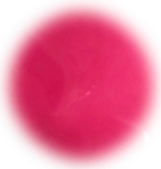	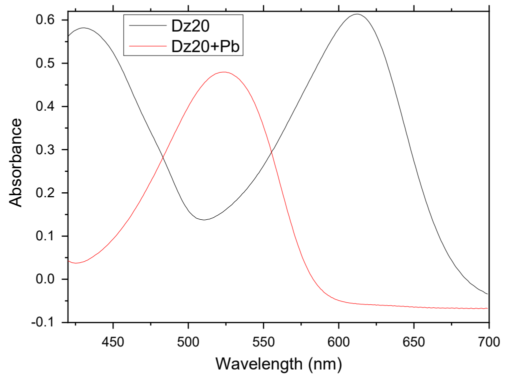

Table S9. Values of the predicted desirability of the multivariate analysis performed with the M2 process method using Dz as chromophore.

**Experimental runs**	**Response**		
	**Expected Desirability**		
	**Dz – Cd(II)**	**Dz – Pb(II)**	**Dz – Hg(II)**
**1**	0.51468	0.393941	0.424206
**2**	0.51468	0.393941	0.424206
**3**	0.463657	0.421779	0.469021
**4**	0.523088	0.504251	0.396784
**5**	0.523088	0.471888	0.638758
**6**	0.51468	0.393941	0.424206
**7**	0.61753	0.618092	0.469021
**8**	0.553152	0.491403	0.45976
**9**	0.553152	0.509909	0.658187
**10**	0.294094	0.058451	0.469021
**11**	0.480357	0.385322	0.45976
**12**	0.480357	0.352591	0.172919
**13**	0.358429	0.51855	0.469021
**14**	0.465424	0.403001	0.474017
**15**	0.465424	0.415281	0.494842
**16**	0.470733	0.46169	0.396784
**17**	0.507994	0.160018	0.41322
**18**	0.51468	0.424416	0.447355
**19**	0.470733	0.417626	0.167815
**20**	0.507994	0.284859	0.48104
**21**	0.51468	0.360902	0.490509
**22**	0.51468	0.393941	0.424206

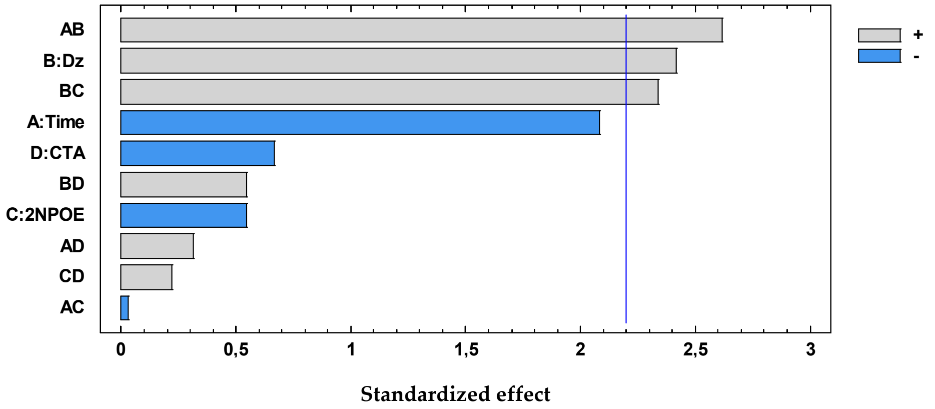

Figure S13. Pareto of the multivariate analysis performed with the M2 process method for the system Dz – Pb(II).

Table S10. ANOVA values of the multivariate analysis performed with the M2 process method for the system Dz – Pb(II).

Source	*Sum of squares*	*Df*	*Mean square*	*F-Ratio*	*P-Value*
**A:Time**	0,0338655	1	0,0338655	4,33	0,0616
**B:Dz**	0,0456172	1	0,0456172	5,83	**0,0343**
**C:2NPOE**	0,00233103	1	0,00233103	0,30	0,5960
**D:CTA**	0,00349265	1	0,00349265	0,45	0,5178
**AB**	0,0535622	1	0,0535622	6,85	**0,0240**
**AC**	0,00000763568	1	0,00000763568	0,00	0,9756
**AD**	0,000762204	1	0,000762204	0,10	0,7608
**BC**	0,0427957	1	0,0427957	5,47	**0,0392**
**BD**	0,0023428	1	0,0023428	0,30	0,5951
**CD**	0,000383735	1	0,000383735	0,05	0,8288
**Total Error**	0,0860455	11	0,00782232		
**Total (corrected)**	0,295103	21			
**R^2**	70,8422 %				
**Adj - R^2**	44,3351 %				
**Standard error**	0,0884439				
**Std. Dev**	0,0459284				

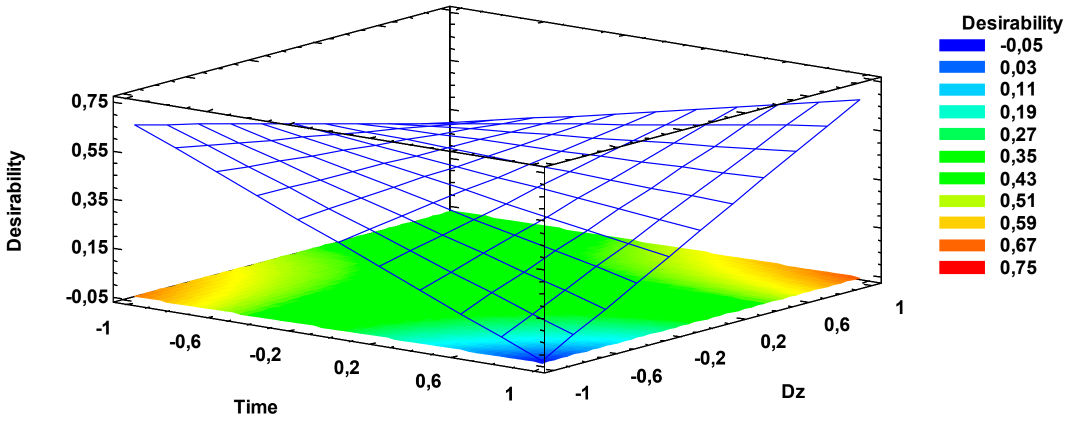

Figure S14. Response surface and contour plots of the multivariate analysis performed with the M2 process method for the system Dz – Pb(II), when CTA = 2NPOE = 0,0.

For the system Dz – Pb(II) the model was:

$$D = 0,401601 + 0,0955195*Dz + 0,267246*Time*Dz - 0,00356558*Time*2NPOE \quad (S7)$$

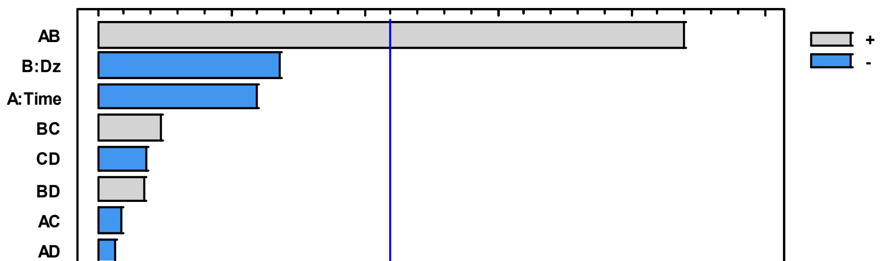

Figure S8. Pareto of the multivariate analysis performed with the M2 process method for the system Dz – Cd(II).

Table S11. ANOVA values of the multivariate analysis performed with the M2 process method for the system Dz – Cd(II).

Source	*Sum of squares*	*Df*	*Mean square*	*F-Ratio*	*P-Value*
**A:Time**	0,00335633	1	0,00335633	1,41	0,2599
**B:Dz**	0,00447673	1	0,00447673	1,88	0,1974
**C:2NPOE**	0,00000180301	1	0,00000180301	0,00	0,9785
**D:CTA**	0,00000108269	1	0,00000108269	0,00	0,9834
**AB**	0,0459382	1	0,0459382	19,32	**0,0011**
**AC**	0,0000720997	1	0,0000720997	0,03	0,8649
**AD**	0,0000400958	1	0,0000400958	0,02	0,8990
**BC**	0,000540699	1	0,000540699	0,23	0,6428
**BD**	0,000286766	1	0,000286766	0,12	0,7350
**CD**	0,000318967	1	0,000318967	0,13	0,7212
**Total Error**	0,0261616	11	0,00237833		
**Total (corrected)**	0,0891673	21			
**R^2**	70,66 %				
**Adj - R^2**	43,9874 %				
**Standard error**	0,0487681				
**Std. Dev**	0,0217078				

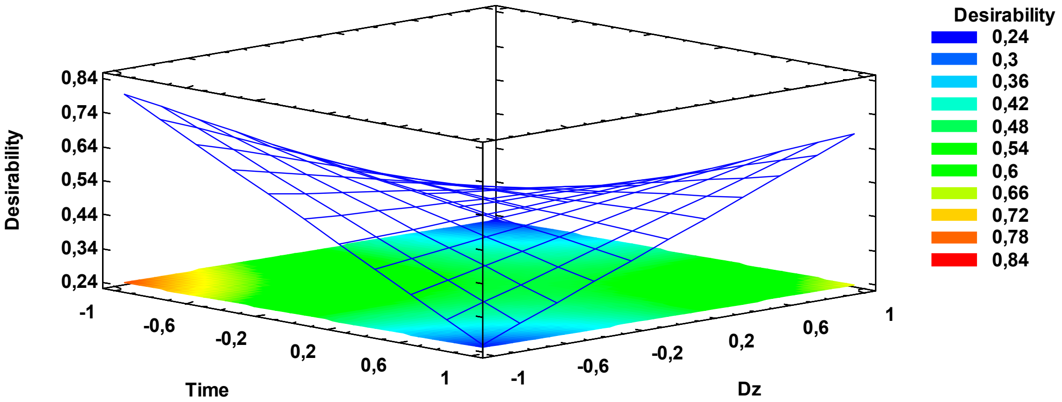

Figure S14. Response surface and contour plots of the multivariate analysis performed with the M2 process method for the system Dz – Cd(II), when CTA = 2NPOE = 0,0.

For the system Dz – Cd(II) the model was:

$$D = 0{,}491962 + 0{,}247497 * Time * Dz \quad (S8)$$

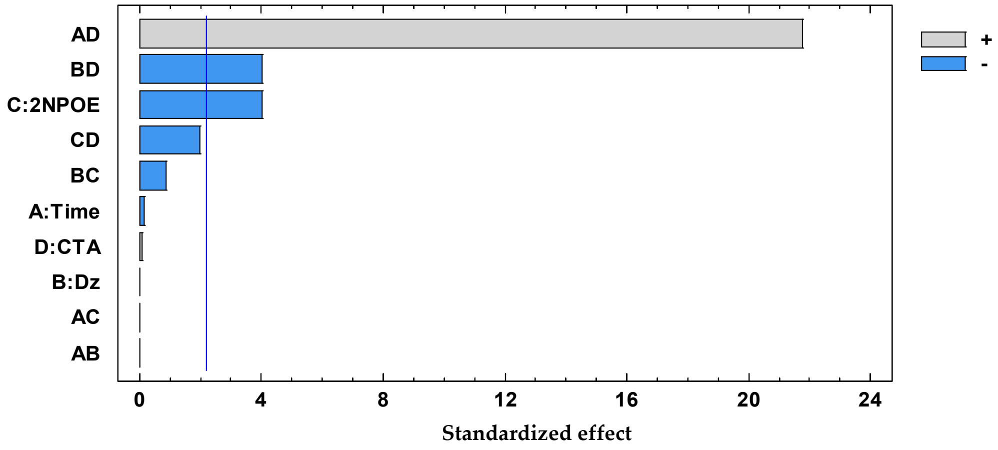

Figure S15. Pareto of the multivariate analysis performed with the M2 process method for the system Dz – Hg(II).

Table S12. ANOVA values of the multivariate analysis performed with the M2 process method for the system Dz – Hg(II).

Source	*Sum of squares*	*Df*	*Mean square*	*F-Ratio*	*P-Value*
**A:Time**	0,0000102603	1	0,0000102603	0,02	0,8799
**B:Dz**	2,76061E-8	1	2,76061E-8	0,00	0,9937
**C:2NPOE**	0,00697371	1	0,00697371	16,26	**0,0020**
**D:CTA**	0,00000281379	1	0,00000281379	0,01	0,9369
**AB**	0,0	1	0,0	0,00	1,0000
**AC**	0,0	1	0,0	0,00	1,0000
**AD**	0,203179	1	0,203179	473,82	**0,0000**
**BC**	0,000314633	1	0,000314633	0,73	0,4099
**BD**	0,00700015	1	0,00700015	16,32	**0,0019**
**CD**	0,00166447	1	0,00166447	3,88	0,0745
**Total Error**	0,00471694	11	0,000428812		
**Total (corrected)**	0,250491	21			
**R^2**	98,1169 %				
**Adj - R^2**	96,405 %				
**Standard error**	0,0207078				
**Std. Dev**	0,0113725				

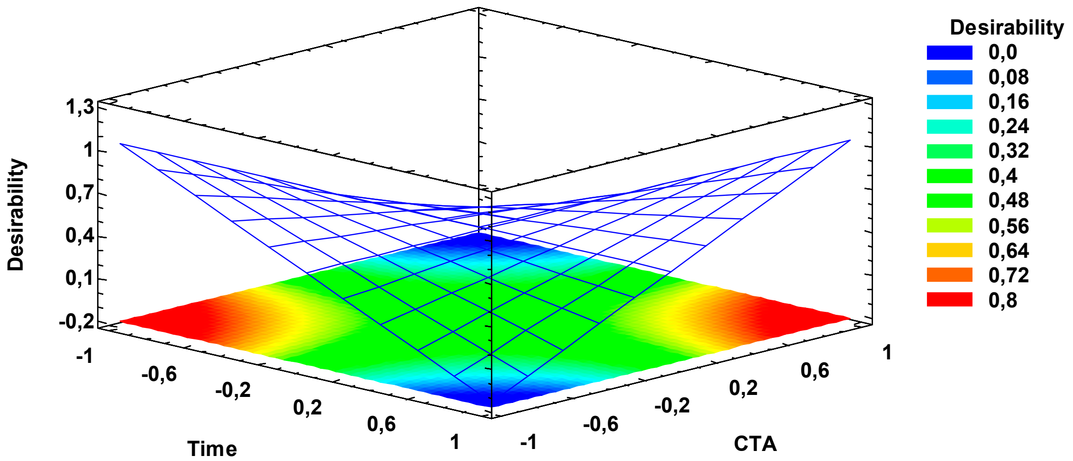

Figure S16. Response surface and contour plots of the multivariate analysis performed with the M2 process method for the system Dz – Hg(II), when Dz = 2NPOE = 0,0.

For the system Dz – Hg(II) the model was:

$$D = 0.442036 - 0.0373232*2NPOE + 0.604432*Time*CTA - 0.109024*Dz*CTA \quad (S9)$$

Table S13. Values of the predicted desirability of the multivariate analysis performed with the M2 process method using PAN as chromophore.

Experimental runs	Response		
	Expected Desirability		
	PAN – Hg(II)	PAN – Pb(II)	PAN – Cd(II)
1	0.543436	0.548901	0.271829
2	0.543436	0.548901	0.271829
3	0.41037	0.613708	0.278323
4	0.521259	0.578848	0.317035
5	0.521259	0.578848	0.317035
6	0.543436	0.548901	0.271829
7	0.503639	0.309131	0.260751
8	0.547868	0.473295	0.312326
9	0.547868	0.473295	0.312326
10	0.503639	0.358989	0.0
11	0.547868	0.505125	0.147643
12	0.547868	0.505125	0.147643
13	0.41037	0.71269	0.135137
14	0.479064	0.64868	0.390385
15	0.479064	0.64868	0.260751
16	0.521259	0.617777	0.229745
17	0.535071	0.426379	0.184666
18	0.543436	0.548901	0.271829
19	0.521259	0.617777	0.229745
20	0.535071	0.426379	0.184666
21	0.543436	0.548901	0.271829
22	0.543436	0.548901	0.271829

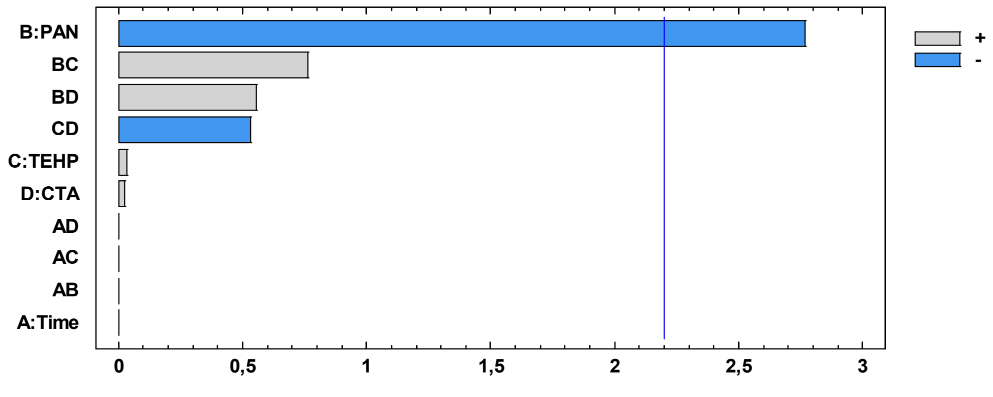

Figure S17. Pareto of the multivariate analysis performed with the M2 process method for the system PAN – Hg(II).

Table S14. ANOVA values of the multivariate analysis performed with the M2 process method for the system PAN – Hg(II).

Source	*Sum of squares*	*Df*	*Mean square*	*F-Ratio*	*P-Value*
**A:Time**	0,0	1	0,0	0,00	1,0000
**B:PAN**	0,013204	1	0,013204	7,65	**0,0183**
**C:THEP**	0,00000174217	1	0,00000174217	0,00	0,9752
**D:CTA**	0,00000104615	1	0,00000104615	0,00	0,9808
**AB**	0,0	1	0,0	0,00	1,0000
**AC**	0,0	1	0,0	0,00	1,0000
**AD**	0,0	1	0,0	0,00	1,0000
**BC**	0,00100899	1	0,00100899	0,58	0,4605
**BD**	0,000535092	1	0,000535092	0,31	0,5887
**CD**	0,000489681	1	0,000489681	0,28	0,6048
**Total Error**	0,018974	11	0,00172491		
**Total (corrected)**	0,0346883	21			
**R^2**	45,3013%				
**Adj - R^2**	0,0%				
**Standard error**	0,0415321				
**Std. Dev**	0,0221346				

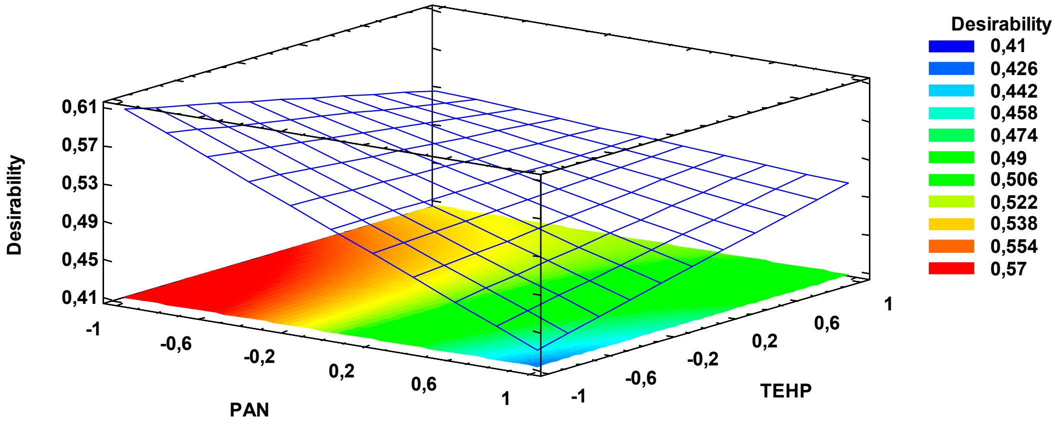

Figure S18. Response surface and contour plots of the multivariate analysis performed with the M2 process method for the system PAN – Hg(II), when CTA = Time = 0,0.

For the system PAN – Hg(II) the model was:

D = 0.517875 + 0.0*Time - 0.0513902*PAN (S10)

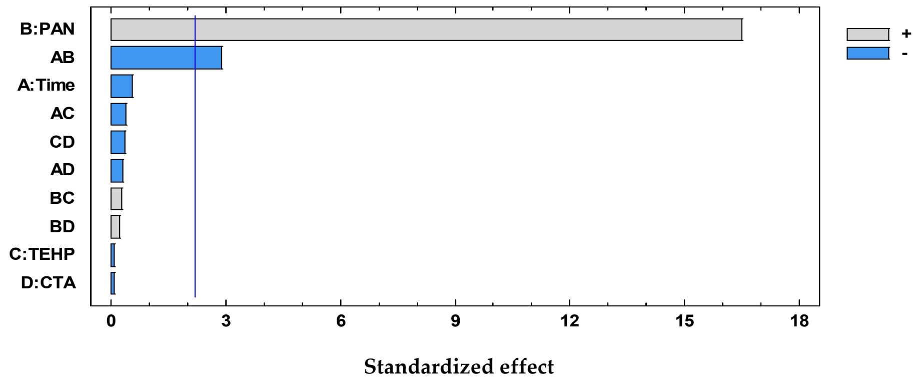

Figure S19. Pareto of the multivariate analysis performed with the M2 process method for the system PAN – Pb(II).

Table S13. ANOVA values of the multivariate analysis performed with the M2 process method for the system PAN – Pb(II).

Source	*Sum of squares*	*Df*	*Mean square*	*F-Ratio*	*P-Value*
**A:Time**	0,000200484	1	0,000200484	0,30	0,5941
**B:PAN**	0,181549	1	0,181549	272,70	**0,0000**
**C:THEP**	0,00000417595	1	0,00000417595	0,01	0,9383
**D:CTA**	0,00000250761	1	0,00000250761	0,00	0,9522
**AB**	0,00553834	1	0,00553834	8,32	**0,0149**
**AC**	0,000100039	1	0,000100039	0,15	0,7057
**AD**	0,0000556332	1	0,0000556332	0,08	0,7779
**BC**	0,0000519484	1	0,0000519484	0,08	0,7852
**BD**	0,0000275653	1	0,0000275653	0,04	0,8425
**CD**	0,0000784508	1	0,0000784508	0,12	0,7379
**Total Error**	0,00732311	11	0,000665738		
**Total (corrected)**	0,197303	21			
**R^2**	96,2884 %				
**Adj - R^2**	92,9142 %				
**Standard error**	0,0258019				
**Std. Dev**	0,0135246				

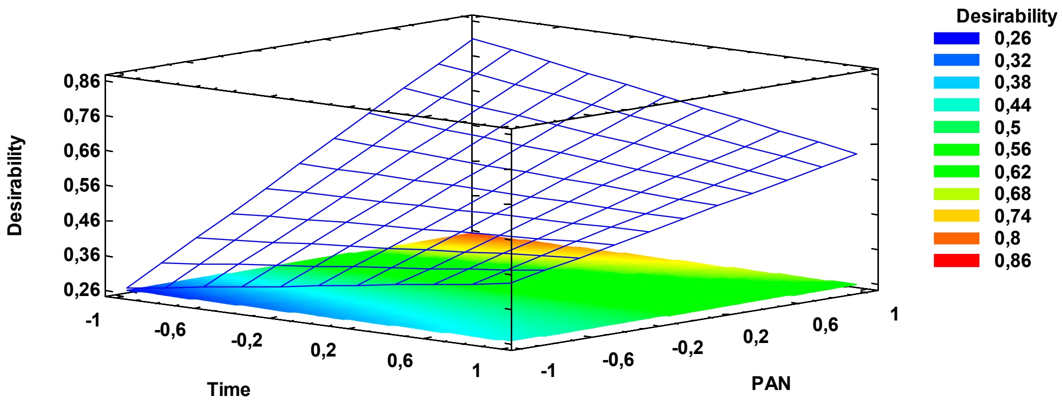

Figure S20. Response surface and contour plots of the multivariate analysis performed with the M2 process method for the system PAN – Pb(II), when CTA = THEP = 0,0.

For the system PAN – Pb(II) the model was:

D = 0.535822 + 0.190557*PAN - 0.0859353*Time*PAN (S11)

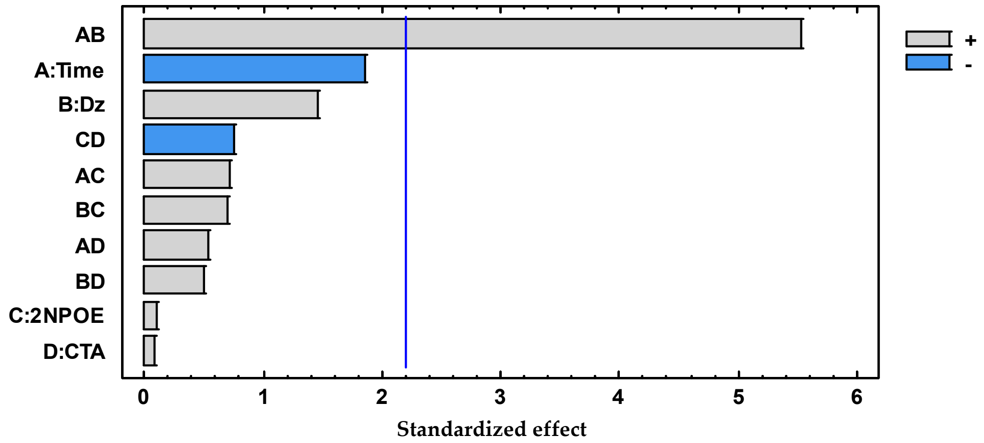

Figure S21. Pareto of the multivariate analysis performed with the M2 process method for the system PAN – Cd(II).

Table S14. ANOVA values of the multivariate analysis performed with the M2 process method for the system PAN – Cd(II).

Source	*Sum of squares*	*Df*	*Mean square*	*F-Ratio*	*P-Value*
**A:Time**	0,0080796	1	0,0080796	3,47	0,0892
**B:PAN**	0,00499023	1	0,00499023	2,15	0,1710
**C:THEP**	0,0000236866	1	0,0000236866	0,01	0,9214
**D:CTA**	0,0000142235	1	0,0000142235	0,01	0,9391
**AB**	0,0711745	1	0,0711745	30,60	**0,0002**
**AC**	0,00122884	1	0,00122884	0,53	0,4825
**AD**	0,000683379	1	0,000683379	0,29	0,5986
**BC**	0,0011196	1	0,0011196	0,48	0,5022
**BD**	0,000594004	1	0,000594004	0,26	0,6233
**CD**	0,00133417	1	0,00133417	0,57	0,4647
**Total Error**	0,0255845	11	0,00232587		
**Total (corrected)**	0,145186	21			
**R^2**	82,3781 %				
**Adj - R^2**	66,3581 %				
**Standard error**	0,0482272				

**Std. Dev**	0,0243175

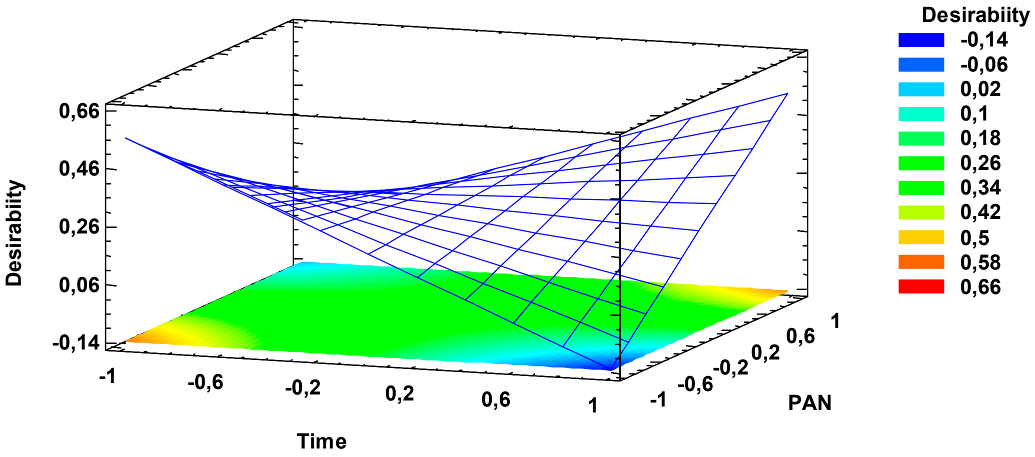

Figure S22. Response surface and contour plots of the multivariate analysis performed with the M2 process method for the system PAN – Cd(II), when CTA = THEP = 0,0.

For the system PAN – Cd(II) the model was:

D = 0.242679 + 0.308066*Time*PAN (S12)

Table S15. Optimization results using the absorbance values before and after complexation.

Optimal experiment	Appearance of the membrane		Spectral
	Before	After	

**PAN + Hg**	8	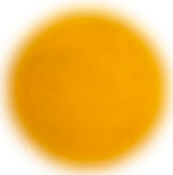	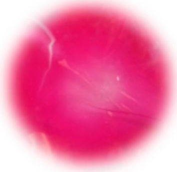	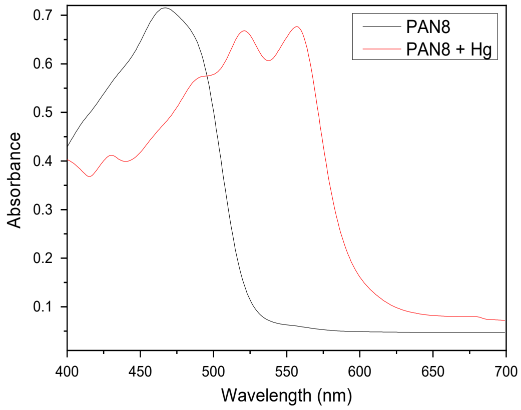
**PAN + Cd**	14	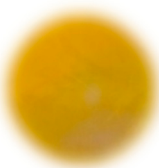	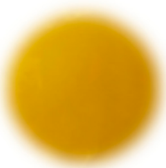	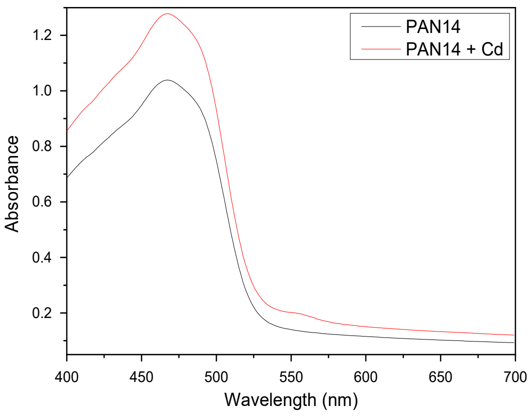
**PAN + Pb**	13	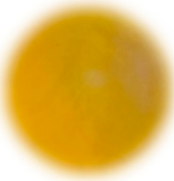	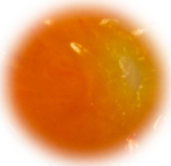	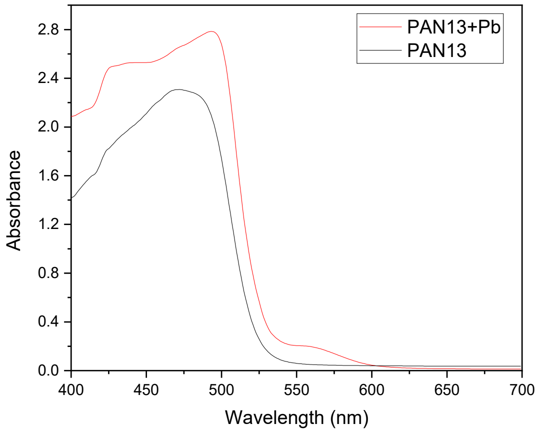
**Dz + Hg**	9	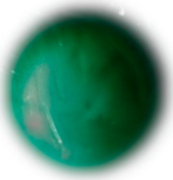	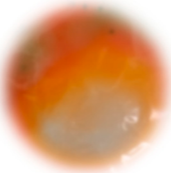	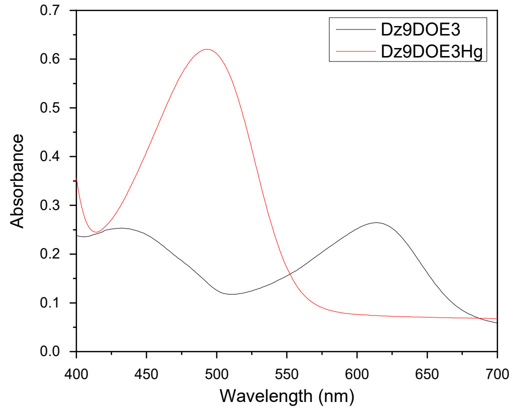

**Dz + Cd**	7	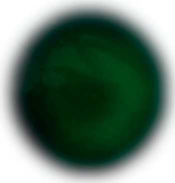	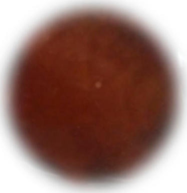	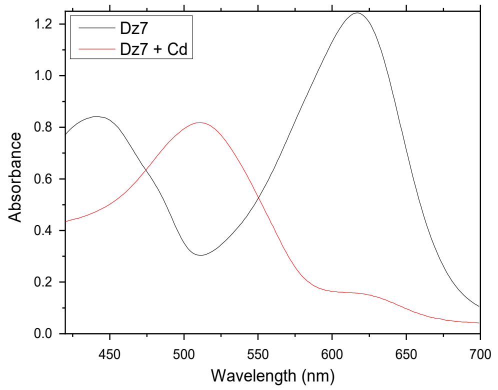
**Dz + Pb**	7	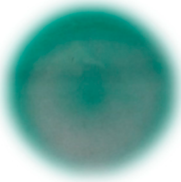	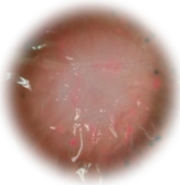	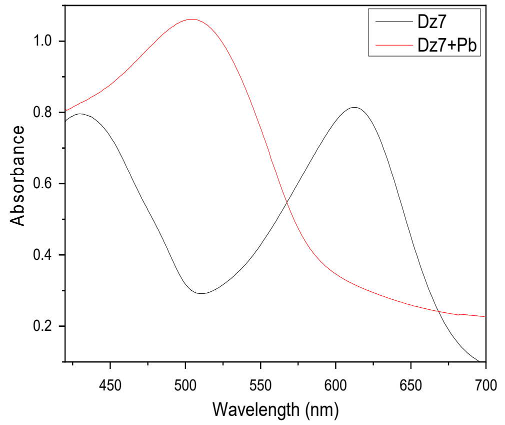

Table S17. Values of the predicted desirability of the multivariate analysis performed with the M3 process method using Dz as chromophore.

Experimental runs	Response		
	Expected Desirability		
	Dz – Cd(II)	Dz – Pb(II)	Dz – Hg(II)
1	0.179692	0.561281	0.685941
2	0.179692	0.561281	0.685941
3	0.269257	0.674154	0.667604
4	0.0	0.601309	0.597549
5	0.0	0.89867	0.896816
6	0.179692	0.561281	0.685941
7	0.494816	0.419034	0.667604
8	0.365697	0.51817	0.597549
9	0.260604	0.46735	0.896816
10	0.324142	0.419034	0.218987
11	0.319801	0.51817	0.708166
12	0.237532	0.194255	0.668952
13	0.176383	0.674154	0.218987
14	0.288784	0.638707	0.592996
15	0.0821848	0.477594	0.700103
16	0.0	0.601309	0.708166
17	0.230607	0.471302	0.592996
18	0.28994	0.401814	0.809836
19	0.0	0.592225	0.668952
20	0.173462	0.63269	0.700103
21	0.331309	0.720747	0.809836
[illegible]	[illegible]	[illegible]	[illegible]

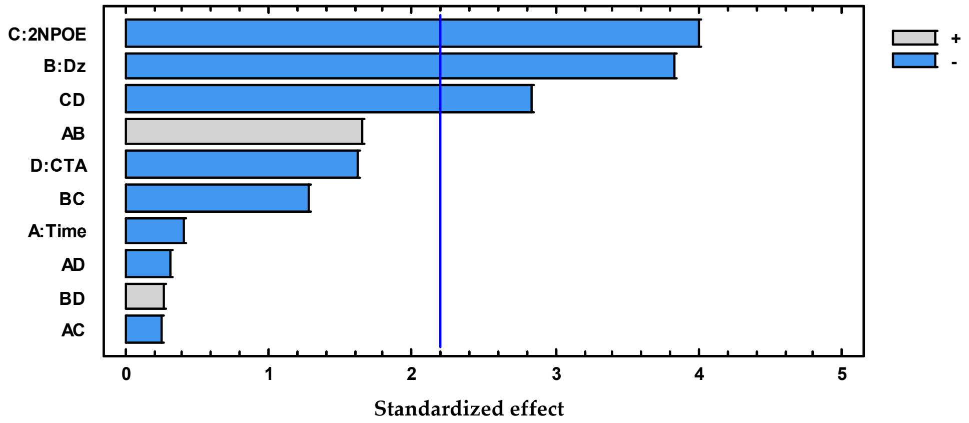

Figure S23. Pareto of the multivariate analysis performed with the M3 process method for the system Dz – Cd(II).

Table S18. ANOVA values of the multivariate analysis performed with the M3 process method for the system Dz – Cd(II).

Source	*Sum of squares*	*Df*	*Mean square*	*F-Ratio*	*P-Value*
**A:Time**	0,00107704	1	0,00107704	0,17	0,6891
**B:Dz**	0,0935983	1	0,0935983	14,66	**0,0028**
**C:2NPOE**	0,102334	1	0,102334	16,03	**0,0021**
**D:CTA**	0,016699	1	0,016699	2,62	0,1341
**AB**	0,0173644	1	0,0173644	2,72	0,1273
**AC**	0,000397844	1	0,000397844	0,06	0,8075
**AD**	0,000657078	1	0,000657078	0,10	0,7543
**BC**	0,0104035	1	0,0104035	1,63	0,2280
**BD**	0,000439125	1	0,000439125	0,07	0,7979
**CD**	0,0513013	1	0,0513013	8,04	**0,0162**
**Total Error**	0,0702125	11	0,00638296		
**Total (corrected)**	0,363744	21			
**R^2**	80,6973 %				
**Adj - R^2**	63,1494 %				
**Standard error**	0,0798934				
**Std. Dev**	0,0418171				

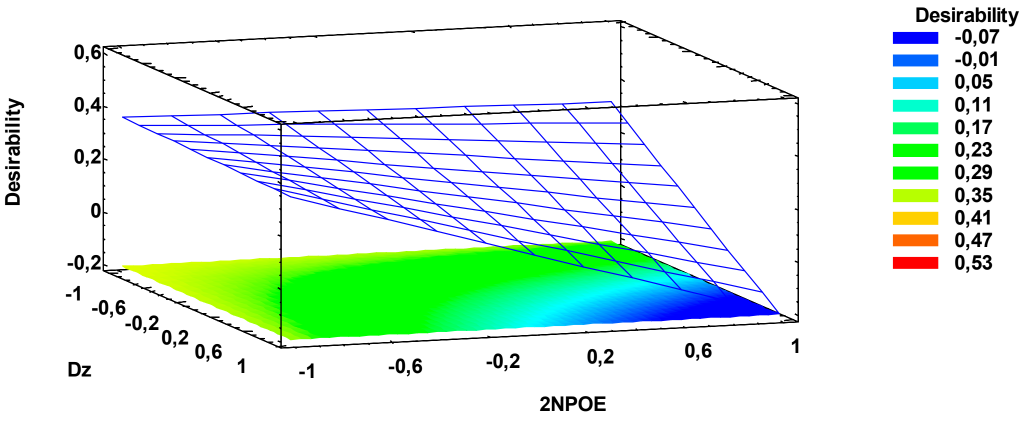

Figure S22. Response surface and contour plots of the multivariate analysis performed with the M3 process method for the system Dz – Cd(II), when CTA = Time = 0,0.

For the system Dz – Cd(II) the model was:

D = 0.207411 - 0.136824*Dz - 0.142974*2NPOE - 0.286167*2NPOE*CTA (13)

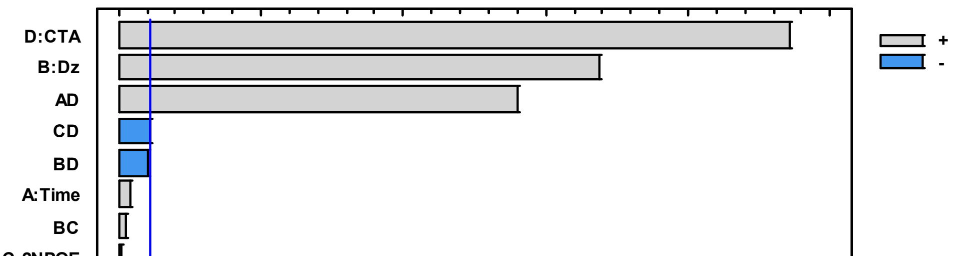

Figure S23. Pareto of the multivariate analysis performed with the M3 process method for the system Dz – Pb(II).

Table S19. ANOVA values of the multivariate analysis performed with the M3 process method for the system Dz – Pb(II).

Source	*Sum of squares*	*Df*	*Mean square*	*F-Ratio*	*P-Value*
**A:Time**	0,0000556111	1	0,0000556111	0,58	0,4610
**B:Dz**	0,108452	1	0,108452	1137,88	**0,0000**
**C:2NPOE**	0,00000389023	1	0,00000389023	0,04	0,8436
**D:CTA**	0,212235	1	0,212235	2226,78	**0,0000**
**AB**	0,0	1	0,0	0,00	1,0000
**AC**	0,0	1	0,0	0,00	1,0000
**AD**	0,0746342	1	0,0746342	783,07	**0,0000**
**BC**	0,0000300068	1	0,0000300068	0,31	0,5860
**BD**	0,000416186	1	0,000416186	4,37	0,0607
**CD**	0,000457735	1	0,000457735	4,80	0,0508
**Total Error**	0,00104841	11	0,0000953102		
**Total (corrected)**	0,406732	21			
**R^2**	99,7422 %				
**Adj - R^2**	99,5079 %				
**Standard error**	0,0097627				
**Std. Dev**	0,00616977				

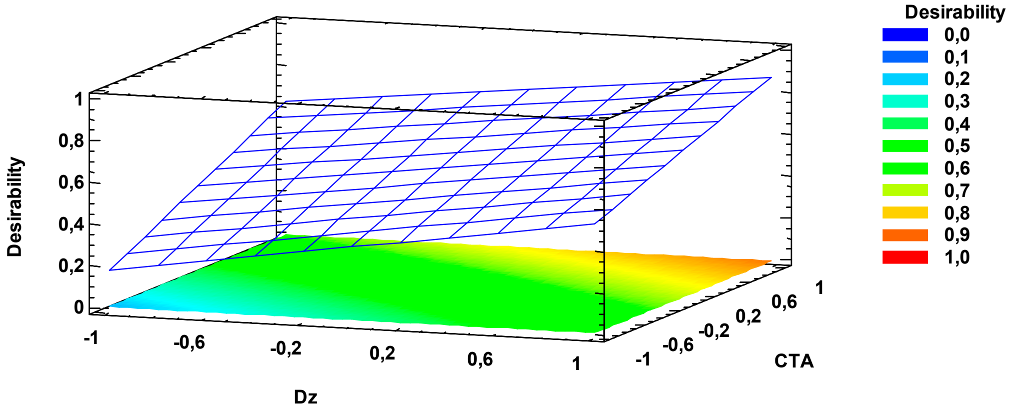

Figure S24. Response surface and contour plots of the multivariate analysis performed with the M3 process method for the system Dz – Pb(II), when 2NPOE = Time = 0,0.

For the system Dz – Pb(II) the model was:

$$D = 0.552991 + 0.147281*Dz + 0.205914*CTA + 0.366334*Time*CTA \quad (S14)$$

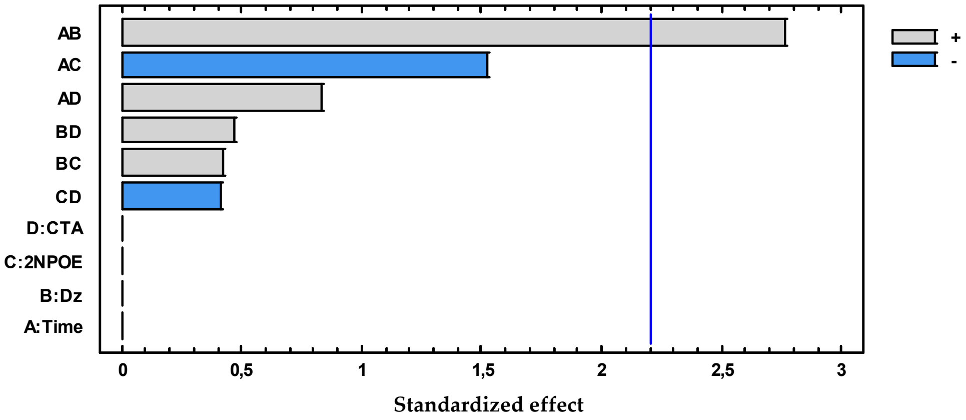

Figure S25. Pareto of the multivariate analysis performed with the M3 process method for the system Dz – Hg(II).

Table S20. ANOVA values of the multivariate analysis performed with the M3 process method for the system Dz – Hg(II).

Source	*Sum of squares*	*Df*	*Mean square*	*F-Ratio*	*P-Value*
**A:Time**	0,0	1	0,0	0,00	1,0000
**B:Dz**	0,0	1	0,0	0,00	1,0000
**C:2NPOE**	0,0	1	0,0	0,00	1,0000
**D:CTA**	0,0	1	0,0	0,00	1,0000
**AB**	0,201257	1	0,201257	7,64	**0,0184**
**AC**	0,0609798	1	0,0609798	2,32	0,1563
**AD**	0,0182156	1	0,0182156	0,69	0,4233
**BC**	0,00477106	1	0,00477106	0,18	0,6786
**BD**	0,00580992	1	0,00580992	0,22	0,6477
**CD**	0,00449788	1	0,00449788	0,17	0,6873
**Total Error**	0,289661	11	0,0263329		
**Total (corrected)**	0,57352	21			
**R^2**	49,4941 %				
**Adj - R^2**	3,57966 %				
**Standard error**	0,162274				
**Std. Dev**	0,0913344				

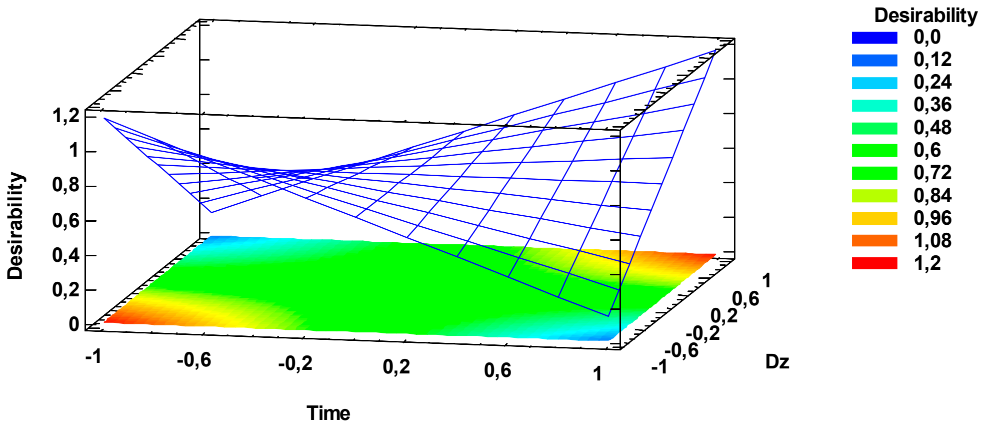

Figure S26. Response surface and contour plots of the multivariate analysis performed with the M3 process method for the system Dz – Hg(II), when 2NPOE = Time = 0,0.

For the system Dz – Hg(II) the model was:

$$D = 0.657514 + 0.518033*Time*Dz \quad (S15)$$

Table S21. Values of the predicted desirability of the multivariate analysis performed with the M3 process method using PAN as chromophore.

Experimental runs	Response		
	Expected Desirability		
	PAN – Cd(II)	PAN – Pb(II)	PAN – Hg(II)
1	0.455591	0.465926	0.416836
2	0.455591	0.223915	0.416836
3	0.430268	0.563115	0.482499
4	0.454742	0.565705	0.401948
5	0.454742	0.392263	0.600185
6	0.455591	0.428557	0.416836
7	0.28149	0.671214	0.433985
8	0.397523	0.643515	0.485418
9	0.397523	0.482728	0.427605
10	0.411835	0.563115	0.433985
11	0.449011	0.565705	0.636049
12	0.449011	0.392263	0.460698
13	0.629506	0.671214	0.482499
14	0.512795	0.261285	0.612344
15	0.512795	0.596423	0.485578
16	0.513641	0.643515	0.574882
17	0.390087	0.261285	0.479722
18	0.455591	0.465926	0.460239
19	0.513641	0.482728	0.646768
20	0.390087	0.596423	0.606119
21	0.455591	0.465926	0.699678
22	0.455591	0.465926	0.416836

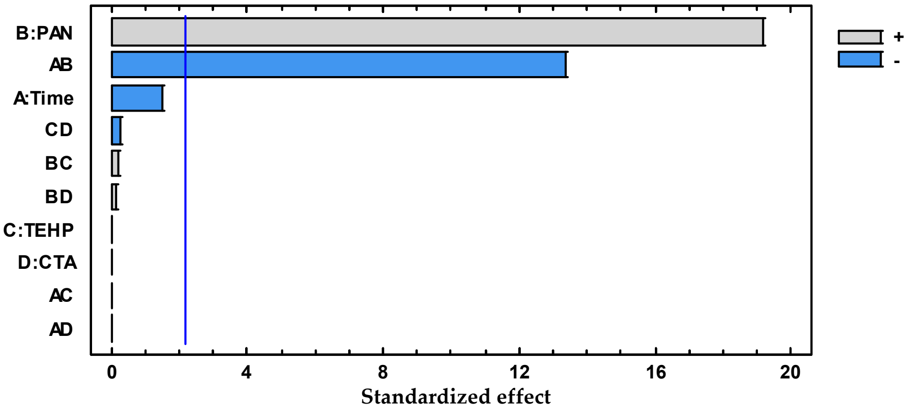

Figure S27. Pareto of the multivariate analysis performed with the M3 process method for the system PAN – Cd(II).

Table S22. ANOVA values of the multivariate analysis performed with the M3 process method for the system PAN – Cd(II).

Source	*Sum of squares*	*Df*	*Mean square*	*F-Ratio*	*P-Value*
**A:Time**	0,00035041	1	0,00035041	2,30	0,1573
**B:PAN**	0,0560517	1	0,0560517	368,35	**0,0000**
**C:THEP**	2,38862E-7	1	2,38862E-7	0,00	0,9691
**D:CTA**	1,43434E-7	1	1,43434E-7	0,00	0,9761
**AB**	0,0271562	1	0,0271562	178,46	**0,0000**
**AC**	3,5837E-8	1	3,5837E-8	0,00	0,9880
**AD**	1,99296E-8	1	1,99296E-8	0,00	0,9911
**BC**	0,00000700214	1	0,00000700214	0,05	0,8341
**BD**	0,00000371548	1	0,00000371548	0,02	0,8787
**CD**	0,0000103725	1	0,0000103725	0,07	0,7989
**Total Error**	0,00167386	11	0,000152169		
**Total (corrected)**	0,0913495	21			
**R^2**	98,1676 %				
**Adj - R^2**	96,5018 %				
**Standard error**	0,0123357				
**Std. Dev**	0,00509949				

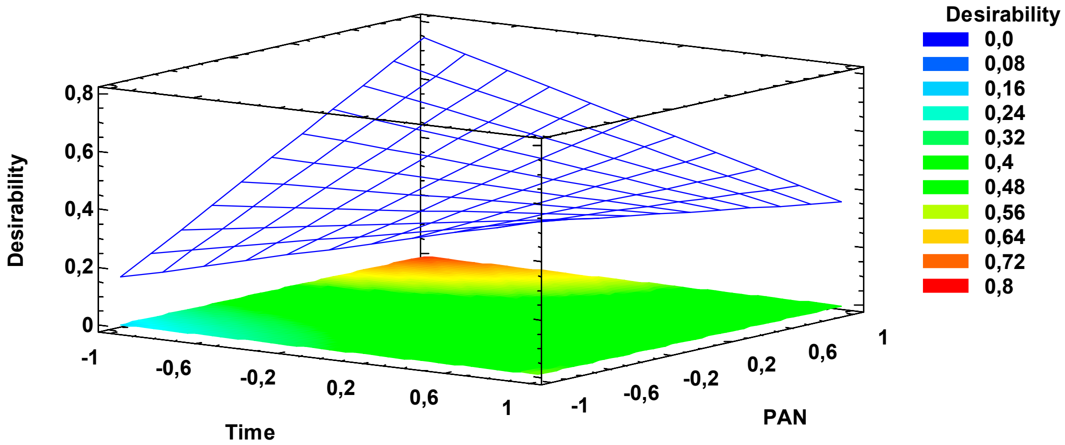

Figure S28. Response surface and contour plots of the multivariate analysis performed with the M3 process method for the system PAN – Cd(II), when THEP = CTA = 0,0.

For the system PAN – Cd(II) the model was:

Desirability= 0.45101 + 0.105882*PAN - 0.19029*Time*PAN (S16)

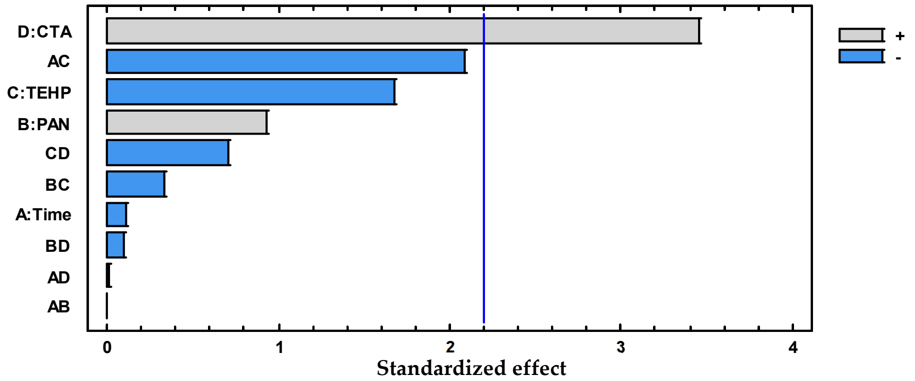

Figure S29. Pareto of the multivariate analysis performed with the M3 process method for the system PAN – Hg(II).

Table S23. ANOVA values of the multivariate analysis performed with the M3 process method for the system PAN – Hg(II).

Source	*Sum of squares*	*Df*	*Mean square*	*F-Ratio*	*P-Value*
**A:Time**	0,0000640569	1	0,0000640569	0,01	0,9152
**B:PAN**	0,00466858	1	0,00466858	0,87	0,3721
**C:THEP**	0,015153	1	0,015153	2,81	0,1218
**D:CTA**	0,0645229	1	0,0645229	11,97	**0,0053**
**AB**	0,0	1	0,0	0,00	1,0000
**AC**	0,0235508	1	0,0235508	4,37	0,0607
**AD**	2,76877E-7	1	2,76877E-7	0,00	0,9944
**BC**	0,000611178	1	0,000611178	0,11	0,7427
**BD**	0,0000548522	1	0,0000548522	0,01	0,9215
**CD**	0,00267146	1	0,00267146	0,50	0,4961
**Total Error**	0,0593142	11	0,0053922		
**Total (corrected)**	0,174981	21			
**R^2**	66.1025 %				
**Adj - R^2**	35,2867 %				
**Standard error**	0,0734316				
**Std. Dev**	0,0479888				

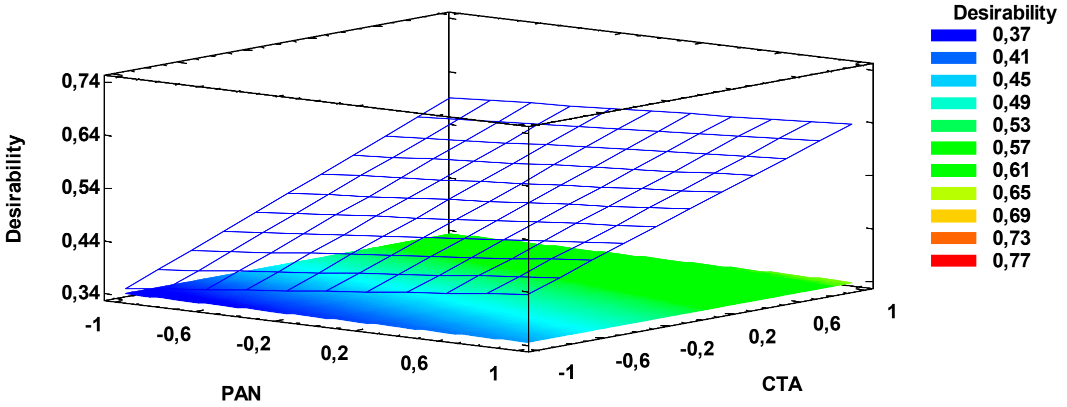

Figure S30. Response surface and contour plots of the multivariate analysis performed with the M3 process method for the system PAN– Hg(II), when THEP = Time = 0,0.

For the system PAN – Hg(II) the model was:

$$D = 0.503524 + 0.113536*CTA \quad (S17)$$

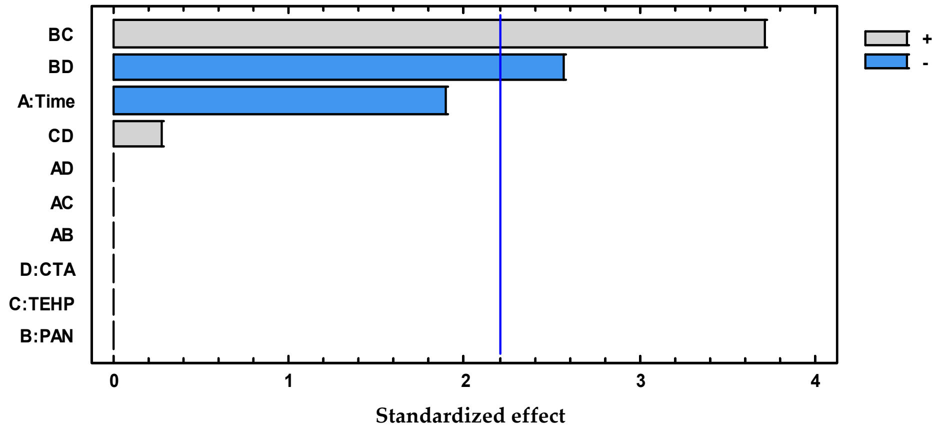

Figure S31. Pareto of the multivariate analysis performed with the M3 process method for the system PAN – Pb(II).

Table S24. ANOVA values of the multivariate analysis performed with the M3 process method for the system PAN – Pb(II).

Source	*Sum of squares*	*Df*	*Mean square*	*F-Ratio*	*P-Value*
**A:Time**	0,0409743	1	0,0409743	3,60	0,0844
**B:PAN**	0,0	1	0,0	0,00	1,0000
**C:THEP**	0,0	1	0,0	0,00	1,0000
**D:CTA**	0,0	1	0,0	0,00	1,0000
**AB**	0,0	1	0,0	0,00	1,0000
**AC**	0,0	1	0,0	0,00	1,0000
**AD**	0,0	1	0,0	0,00	1,0000
**BC**	0,157157	1	0,157157	13,80	**0,0034**
**BD**	0,0751327	1	0,0751327	6,60	**0,0261**
**CD**	0,000909381	1	0,000909381	0,08	0,7827
**Total Error**	0,125266	11	0,0113878		
**Total (corrected)**	0,357986	21			
**R^2**	65,0082 %				
**Adj - R^2**	33,1974 %				
**Standard error**	0,106714				

**Std. Dev**	0,0468218

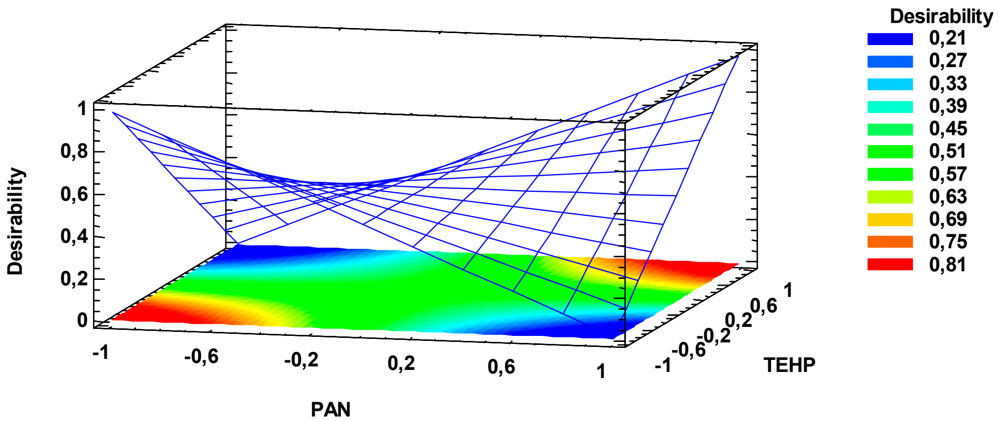

Figure S32. Response surface and contour plots of the multivariate analysis performed with the M3 process method for the system PAN – Pb(II), when CTA = Time = 0,0.

For the system PAN – Pb(II) the model was:

D = 0.494014 - + 0.48524*PAN*TEHP - 0.357177*PAN*CTA (S18)
